# Inequality in fertility rate among adolescents: evidence from Timor-Leste demographic and health surveys 2009–2016

**DOI:** 10.1186/s13690-020-00484-1

**Published:** 2020-10-14

**Authors:** Sanni Yaya, Betregiorgis Zegeye, Bright Opoku Ahinkorah, Kelechi Elizabeth Oladimeji, Gebretsadik Shibre

**Affiliations:** 1 School of International Development and Global Studies, University of Ottawa, Ottawa, Canada; 2 The George Institute for Global Health, Imperial College London, London, UK; 3 HaSET Maternal and Child Health Research Program, Shewarobit Field Office, Shewarobit, Ethiopia; 4 The Australian Centre for Public and Population Health Research (ACPPHR), Faculty of Health, University of Technology Sydney, Sydney, Australia; 5 Department of Public Health, Faculty of Health Sciences, University of Fort Hare, Eastern Cape, South Africa; 6 Center for Community Healthcare, Research and Development, Benin-City, Nigeria; 7 Department of Reproductive, Family and Population Health, School of Public Health, Addis Ababa University, Addis Ababa, Ethiopia

**Keywords:** Adolescent fertility, Inequality, Global health, Timor-Leste, Demographic and health surveys

## Abstract

**Background:**

Despite a decline in global adolescent birth rate, many countries in South East Asia still experience a slower pace decline in adolescent birth rates. Timor-Leste is one of the countries in the region with the highest adolescent birth rate and huge disparities between socio-economic subgroups. Hence, this study assessed the magnitude and trends in adolescent fertility rates within different socio-demographic subgroups in Timor-Leste.

**Methods:**

Using the World Health Organization’s (WHO) Health Equity Assessment Toolkit (HEAT) software, data from the Timor-Leste Demographic and Health surveys (TLDHS) were analyzed between 2009 and 2016. We approached the inequality analysis in two steps. First, we disaggregated adolescent fertility rates by four equity stratifiers: wealth index, education, residence and region. Second, we measured the inequality through summary measures, namely Difference, Population Attributable Risk, Ratio and Population Attributable Fraction. A 95% confidence interval was constructed for point estimates to measure statistical significance.

**Results:**

We found large socio-economic and area-based inequalities over the last 7 years. Adolescent girls who were poor (Population Attributable Fraction: -54.87, 95% CI; − 57.73, − 52.02; Population Attributable Risk: -24.25, 95% CI; − 25.51, − 22.99), uneducated (Difference: 58.69, 95% CI; 31.19, 86.18; Population Attributable Fraction: -25.83, 95% CI; − 26.93, − 24.74), from rural areas (Ratio: 2.76, 95% CI; 1.91, 3.60; Population Attributable Risk: -23.10, 95% CI; − 24.12, − 22.09) and from the Oecussi region (Population Attributable Fraction: -53.37, 95% CI; − 56.07, − 50.67; Difference: 60.49, 95% CI; 29.57, 91.41) had higher chance of having more births than those who were rich, educated, urban residents and from the Dili region, respectively.

**Conclusions:**

This study identified disproportionately higher burden of teenage birth among disadvantaged adolescents who are, poor, uneducated, rural residents and those living in regions such as Oecussi, Liquica and Manufahi, respectively. Policymakers should work to prevent child marriage and early fertility to ensure continuous education, reproductive health care and livelihood opportunities for adolescent girls. Specialized interventions should also be drawn to the subpopulation that had disproportionately higher adolescent childbirth.

## Background

Globally, there are about 1.2 billion adolescents aged 10–19 years, accounting 16% of the world’s population today [1]. There are 340 million adolescents in South Asia, which accounts for approximately 30% of adolescents in the globe [2]. Even though adolescents contribute largely to the population of South Asia, they are often largely invisible and ignored [2]. Adolescent pregnancies remain serious problems in many countries. Globally, approximately 16 million adolescents aged 15–19 years become pregnant each year [3].

In low-and middle-income countries, an estimated 21 million girls aged 15–19 become pregnant every year [3] and nearly 12 million 15–19 years girls and 777,000 younger than 15 years adolescent give birth respectively [3]. Though there has been recent global decline in Adolescent Fertility Rate (AFR) [4],defined as the number of births per 1000 women aged 15 to 19 years [5], these rates are either stagnant or increasing in several countries in East Asia and the Pacific [6]. In the South East Asia region, AFR remains high at 47 for the period 2007–2016 [6] compared to a global average of 44 per 1000 women [7]. The adolescent pregnancy rates across South East Asia is approximately 94.4 in Lao People’s Democratic Republic, 41.9 in Timor-Leste and 2.7 in Singapore [8]. While it is assumed that most adolescent pregnancies result from inadequate sex education, lack of access to contraception and reproductive health services [6, 9], recent studies show that there are many complex socio-economic and cultural factors that can increase the likelihood of adolescent pregnancy [10, 11].

Maternal and neonatal deaths linked to pregnancy and childbirth-related complications are one of the undesirable outcomes of adolescent pregnancies [12, 13]. Evidence shows that there are approximately 3.9 million adolescent girls exposed to unsafe abortion each year, which significantly contributes to maternal morbidity and mortality [3, 12]. Moreover, adolescent mothers aged 10–19 face life-threatening risks from pregnancy-induced hypertension, puerperal endometritis and sepsis as compared to mothers in older age groups [13, 14]. Adolescent pregnancy is also associated with premature delivery, stillbirth, fetal distress, asphyxia at birth, low birth weight, and miscarriage [3, 14]. In addition, many of these “teenage mothers” are unmarried, never planned for their pregnancies and because of the prevailing stigma associated with adolescent pregnancies they may choose not to visit a healthcare facility for care [3, 15]. Lastly, adolescent pregnancy is associated with increased school dropout rates which worsen their economic circumstances [3, 16].

In previous studies, socioeconomic factors, such as low education and low income; inadequate sex education; lack of access to contraceptives and reproductive health services were considered as predictors of adolescent pregnancies. Another predisposing factor identified for adolescent pregnancy is living in rural areas where harmful cultural norms and practices hold way [10, 11, 17, 18].

Timor-Leste has a considerable “youth bulge” as the majority of the population in the country is under the age of 25 years. The country is therefore ranked seventh in the world as the youngest country. Despite this, deaths among adolescents due to pregnancy-related complication is high, with 789 deaths per 100,000 live births documented among women aged 15–19 [9, 19, 20]. Nearly 7% of adolescent girls aged 15–19 have begun childbearing—5% are mothers and 2% are pregnant with their first child. Teenage fertility is twice as high in rural areas (8%) compared to in urban areas (4%). By municipality, teenage fertility ranges from 3% in Dili to 10% in Bobonaro and SAR of Oecussi [9].

Gender norms and unequal power relationships between girls and boys are implied drivers of adolescent pregnancy in Timor-Leste [9]. High-quality evidence on the state of inequality around AFR is critical to the planning of equity-sensitive interventions to improve adolescent girls’ health. As far as Timor-Leste is concerned, no study has examined this issue to the best of our knowledge. Accordingly, this study examined the magnitude of socio-economic and area-based disparities in adolescent fertility rate in Timor-Leste as well as the trends of within-country inequality over the last 7 years.

## Methods

### Study setting

Timor-Leste is one of the lower-middle-income countries in Southeast Asia and has a population density of 1.2 million that is growing at 3.2% per year [21]. Majority of the population in the country live in rural areas (74%) and are engaged in small-scale subsistence farmers [22]. In line with its ethnic mix of colonial history [23], the country has twelve ethnic groups [23], with thirty-two [24] number of languages spoken, of which Tetum and Portuguese remain official languages, while Indonesian and English are working languages [25, 26].

Oil is the major source of revenue for the country and it accounted for more than 30% of the country’s total revenue in 2016 [27]. Between 2007 and 2012, there was fluctuations in headline economic growth rates leading to more than doubling in GNI per capita. This was due to variations in oil prices and falling production. However, the economic growth dropped back to its 2007 level by 2016. Non-oil GDP per capita has risen steadily, growing an average 5% a year from 2006 to 2016 when it stood at US$1336 per person [27]. In 2018, gross national income was US$ 7658 per capita and gross domestic product growth was 4% per year (dollars are valued at purchasing power parity [21]. The discovery of oil brought advancement in the economy and improved capital development, including reinforcement of the health care system [28, 29]. Consequently, the healthcare system in Timor-Leste is operated by the public sector through public funding.

In the country, there is cost-free health services provision at the point of use, through the large proportionate government contributions to expenditure on health care (90% of total health care expenditure) [28]. The country operates a three-tier health care delivery system, in which the National Hospital in Dili (the capital) provides tertiary care, 5 referral hospitals at the district level provide secondary services, and a network of 66 community health centers (CHCs) and 205 health posts deliver primary health care (PHC) services across the country’s 13 districts. In addition, the CHCs carry out special monthly outreach programmes known locally as *Servisu Integrado du Saude Comunidade* (SISCa) [30]. To ensure adequate access to healthcare services, health services have been designed in such a way that everyone should have access to them within a 1hour walking distance. The private health system remains relatively underdeveloped, although the Ministry of Health (MoH) estimates that about 25% of basic health services are delivered by private providers (both for profit and non-profit) [30].

### Data source and study population

The Demographic and Health Surveys conducted in Timor-Leste in 2009 and 2016 were used as the sources of data for this study. Briefly, a two-stage cluster design was employed to select women aged 15 to 49 years, men aged between 15 to 59 years, and children. The DHS surveys are nationally representative and collect data on a wide range of public health related topics and indicators such as maternal health services, child health, maternal and childhood mortality, socioeconomic status, family planning and domestic violence. These surveys were carried out with the financial and technical assistance of Inner-City Fund (ICF) International and provisioned through the USAID-funded MEASURE DHS program. The survey also enrolled adolescent girls aged 15 to 19 years. The detailed methodology of the TDHS is available in the surveys' final reports [24, 31].

### Measures of inequality

The inequality variable measured in this study is adolescent fertility rate (AFR). It is measured as proportion of births per 1000 women aged 15–19. We disaggregated the AFR by four equity stratifiers: economic status, education, subnational region and place of residence. We approximated economic status through a composite variable known as wealth index. In the DHS, wealth index is computed using different household ownerships and characteristics following Principal Component Analysis (PCA) technique [32].

Wealth index has five categories: poorest, poor, middle, rich and richest. Educational status of the woman was classified as no-education, primary and secondary/higher and place of residence as urban versus rural. The disaggregated AFR (reported as percentage) was presented for each of the four Timor-Leste Demographic and Health Surveys (TLDHS) time periods. The educational status and wealth index have a natural ordering and are known as ordered equity stratifiers whereas region is a non-ordered equity stratifier. Whether an equity stratifier is ordered or not, affects the choice of summary measures to be calculated [33].

### Statistical analysis

We used the 2019 updated version of the World Health Organization’s (WHO) Health Equity Assessment Toolkit (HEAT) software [33] for analyzing the socioeconomic and area-based inequalities associated with AFR. Detailed description of the software has been given elsewhere [33]. The WHO released the software in 2016 through the use of free and publicly available R programming language and the R packages. The software provides the opportunity to do assessment of within country health inequalities of more than 30 indicators for reproductive, maternal, newborn, and child health (RMNCH). It also creates room for benchmarking inequality in one country with that of another country, allowing for direct comparison of inequality in two or more countries at the same time [33, 34]. The type of health indicator of interest (favorable versus adverse) and the inherent properties of dimensions of inequality determined the choice and interpretations of summary measures for this study. We computed the summary measures with the mindset that AFR is an adverse health indicator.

We carried out our data analyses using a two-step approach. First, we disaggregated AFR by the commonly used dimensions of inequality (economic status, education, subnational region and place of residence). Then, we calculated summary measures and used a combination of absolute and relative inequality summary measures. These were Difference (D), Population Attributable Risk (PAR), Population Attributable Fraction (PAF) and Ratio (R). The first two (D and PAR) are absolute inequality measures while the other two (PAF and R) are relative measures of inequality. These measures were calculated for each of the four equity stratifiers. The detailed methods of calculation, interpretation and all other detailed properties of the measures employed in the study have been described elsewhere in detail [33].

The R and D are simple measures suitable for determining the relative ratio and absolute difference between two categories within a dimension of inequality (i.e. urban versus rural for residence) or between 2 or more categories (i.e. regions) based on an identified reference subgroup in the category. PAR and PAF are weighted complex measures of inequality that consider the sizes of subpopulations used in the calculation, hence producing estimates reflective of the subpopulation size [33, 34]. Complex measures are the ones that take into consideration the sizes of the subpopulation used in the calculation of a measure in question, thereby producing estimates reflective of size of the subpopulation [33, 34].

The PAF and PAR take positive values for favorable health intervention indicators and negative values for adverse health outcome indicators like AFR. Zero shows absence of inequality and the greater absolute value of PAF and PAR indicates a higher level of inequality. PAR is calculated as the difference between the subgroup with the lowest estimate and the national average of the indicator for adverse outcome indicators. For ordered dimensions like wealth and education, PAR is the difference between the most-advantaged subgroup and the national average, regardless of the indicator type. PAF is calculated by dividing the PAR by the national average μ and multiplying the fraction by 100: PAF = [PAR / μ] * 100.

For binary dimensions like residence, difference is calculated as the difference between the subgroup with the highest estimate (rural) and the subgroup with the lowest estimate (urban), regardless of the indicator type. For ordered dimensions like wealth and education, it is the difference between the most-disadvantaged subgroup and the most advantaged subgroup. For binary dimensions like residence, ratio is calculated as the difference between the subgroup with the highest estimate (rural) and the subgroup with the lowest estimate (urban), regardless of the indicator type. For ordered dimensions like wealth and education, it is the Ratio between the most-disadvantaged subgroup and the most advantaged subgroup. In the absence of inequality, Difference and Ratio become zero and one, respectively. Point estimates were calculated and presented with corresponding 95% Confidence Intervals. To examine whether AFR shows statistically significant disparities across the sub-groups of each equity stratifier, and to determine whether or not the inequality changed with time, we computed 95% Confidence Intervals (CI) around point estimates of each measure for each survey. For all inequality measures other than Ratio, the lower and upper bounds of the CI must not include zero to interpret that inequality exists. For Ratio, the interval should not include one. We assessed the trend of inequality for each summary measure by referring to the CIs for the different survey years; if the CIs did not overlap, inequality existed.

### Ethical consideration

This study used publicly available data stored in the HEAT software application. Informed consent for the TDHS that were available in the HEAT software was obtained from participants prior to the survey. The DHS Program follows ethical standards for ensuring the protection of respondents’ privacy. ICF International ensures that the survey complies with the U.S. Department of Health and Human Services regulations for the respect of the right of human subjects. No further approval was required for this study since the data is secondary and available in the public domain. More details about DHS data and ethical standards are available at: https://bit.ly/2XHjJsR

## Results

This study involved a total of 26, 234 population from both 2009 and 2016 surveys. Of them, 17,736 (67.6%) were rural residents and 3880 (14.8%) were from poorest wealth quintile. Regarding educational status, nearly three fourth (74.6%) of the participants had secondary school and above education, whereas, 3686 (14%) and 2964 (11.3%) had primary and no formal education, respectively. Five regions accounted for more than half (53.7%) of the participants, which were Dili (23.8%), Ermera (10.9%), Baucau (10.5%), and Bobonaro (8.5%).

Table 1 shows the magnitude and trends of AFR across socio-economic, urban-rural and subnational subpopulations in Timor-Leste from 2009 to 2016. The results revealed disproportionately higher AFR among the disadvantaged subgroups. The extent of AFR varied across wealth quintiles with higher concentration among those in the poorest and middle wealth quintile. In 2009, the highest AFR was observed among adolescents in the middle wealth quintile and poorest wealth categories, while in 2016, highest proportion was in the poorest and poorer wealth quintiles. In both 2009 and 2016, the proportions of the study population with no formal education had the highest AFR (Table 1).

**Table 1:** Adolescent fertility rate disaggregated across socio-economic, urban-rural and subnational subpopulations in Timor-Leste using data from the 2009–2016 Timor-Leste Demographic and Health Surveys

Dimension of Inequality	Subgroup	2009		2016	
		Estimate (95% CI)	Population	Estimate (95% CI)	Population
Economic status	Poorest quintile	62.61 (50.83, 76.90)	2134	80.20 (60.50, 105.58)	1746
	Poorer quintile	58.03 (45.97, 73.01)	2574	66.99 (52.88, 84.54)	2217
	Middle quintile	70.56 (56.95, 87.12)	2764	47.88 (35.97, 63.47)	2347
	Richer quintile	50.77 (39.26, 65.41)	3022	29.90 (22.09, 40.34)	3002
	Richest quintile	35.35 (24.63, 50.50)	3159	19.94 (13.41, 29.54)	3264
Education	No education	73.90 (59.85, 90.94)	1806	91.46 (67.84, 122.23)	1158
	Primary school	105.50 (86.83, 127.62)	2633	104.55 (76.18, 141.85)	1053
	Secondary school +	36.02 (30.30, 42.77)	9216	32.77 (27.63, 38.84)	10,366
Place of residence	Rural	58.66 (53.02, 64.85)	9905	58.20 (50.82, 66.58)	7831
	Urban	43.26 (34.57, 54.02)	3750	21.08 (15.99, 27.75)	4748
Subnational region	01 Aileu	54.99 (41.34, 72.81)	656	31.32 (21.20, 46.04)	578
	02 Ainaro	59.62 (41.74, 84.49)	645	44.87 (26.19, 75.83)	461
	03 Baucau	58.62 (43.22, 79.07)	1395	45.53 (28.15, 72.83)	1355
	04 Bobonaro	63.81 (50.50, 80.33)	1464	67.47 (46.46, 97.02)	767
	05 Cova lima	49.67 (35.57, 68.96)	836	65.46 (38.52, 109.08)	665
	06 Dili	42.92 (31.45, 58.30)	2603	20.60 (14.47, 29.24)	3647
	07 Ermera	48.62 (33.74, 69.58)	1665	35.97 (23.46, 54.78)	1193
	08 Liquica	47.79 (33.59, 67.55)	888	36.72 (23.05, 58.03)	625
	09 Lautem	62.87 (45.30, 86.64)	801	76.99 (56.97, 103.29)	824
	10 Manufahi	60.10 (43.32, 82.80)	498	76.64 (48.14, 119.89)	448
	11 Manatuto	54.42 (39.07, 75.33)	661	51.31 (35.05, 74.52)	735
	12 Oecussi	81.12 (62.22, 105.11)	742	81.09 (55.65, 116.74)	537
	13 Viqueque	50.43 (34.35, 73.46)	796	53.94 (35.90, 80.30)	739

### Trends of AFR indicating the impact of inequality

Table 2 shows the socio-economic, urban-rural and subnational subpopulations disparities in AFR between 2009 and 2016, favoring adolescents who are socio-economically better-off, urban residents and those living in regions like Dili.

**Table 2:** Trends of socio-economic and area-based inequality in adolescent fertility rate in Timor-Leste using data from the 2009–2016 Timor-Leste Demographic and Health Surveys

Dimension	Measure	2009	2016	Trend from 2009 to 2016
		%(95%CI)	%(95%CI)	
Economic status	D	27.26 (9.13, 45.39)	60.25 (36.59, 83.92)	Increased
	PAF	−35.05 (−37.80, −32.30)	−54.87 (−57.73, −52.02)	Increased
	PAR	−19.08 (−20.57, −17.58)	−24.25 (−25.51, −22.99)	Increased
	R	1.77 (1.03, 2.50)	4.02 (2.08, 5.96)	Increased
Education	D	37.88 (21.23, 54.52)	58.69 (31.19, 86.18)	Constant
	PAF	−33.81 (−34.99, −32.64)	−25.83 (−26.93, −24.74)	Decreased
	PAR	−18.40 (−19.04, −17.76)	−11.41 (−11.90, −10.93)	Decreased
	R	2.05 (1.49, 2.60)	2.79 (1.84, 3.73)	Constant
Residence	D	15.39 (4.07, 26.70)	37.12 (27.35, 46.88)	Decrease
	PAF	−20.51 (−23.01, −18.00)	−52.28 (−54.58, −49.98)	Increased
	PAR	−11.16 (−12.52, −9.80)	−23.10 (−24.12, −22.09)	Increased
	R	1.35 (1.02, 1.68)	2.76 (1.91, 3.60)	Increased
Region	D	38.20 (13.15, 63.24)	60.49 (29.57, 91.41)	Constant
	PAF	−21.15 (−24.31, −17.98)	−53.37 (−56.07, −50.67)	Increased
	PAR	−11.51 (−13.23, −9.79)	−23.59 (− 24.78, −22.39)	Increased
	R	1.89 (1.12, 2.65)	3.93 (1.92, 5.94)	Increased

Substantial absolute and relative wealth-driven inequality in AFR were observed from 2009 to 2016 both by simple (D, R) and complex (PAF, PAR) measures. Increasing economic disparity pattern was also seen. For instance, in the 2016 survey, the PAR measure (PAR = -24.25, 95% CI; − 25.51, − 22.99) and the PAF measure (PAF = − 54.87, 95% CI; − 57.73, − 52.02) respectively, signified significant absolute and relative economic related inequality favoring economically better off subpopulations respectively.

We found significant absolute (PAR = − 11.41, 95% CI; − 11.90, − 10.93) and relative (PAF = -25.83, 95% CI; − 26.93, − 24.74) education-based inequality in AFR on both surveys’ points using simple (D, R) and complex (PAR) measures with higher burden AFR among disadvantaged subpopulation (not educated). Its pattern was constant by simple measures (D, R), while complex measures (PAR, PAF) suggested decreasing.

We noticed absolute and relative urban-rural inequality in AFR from 2009 to 2016 both by simple (D, R) and complex (PAR, PAF) measures with an increasing pattern. For instance, the Ratio measure (*R* = 2.76, 95% CI; 1.91, 3.60) in 2016 survey, tell us huge relative pro-urban disparities in AFR with over time increasing pattern.

Our finding also shows absolute (D, PAR) and relative (R, PAF) inequality in AFR across subnational region, over the last 7 years. Nevertheless, it seemed constant by simple measures (D, R), the regional disparities becoming increased by complex measures (PAR, PAF). For example, in 2016 survey, The Difference measure (D = 60.49, 95% CI; 29.57, 91.41) and the PAF measure (PAF = -53.37, 95% CI; − 56.07, − 50.67) indicating substantial absolute and relative regional inequality (Table 2).

## Discussion

This study reported the extent and change of inequality in adolescent fertility rate (AFR) between 2009 and 2016 in Timor-Leste. The study confirmed that socioeconomic and area-based inequalities in the time intervals assessed, using epidemiological summary measures and equity stratifiers, had a significant influence on AFR. Over the seven-year study period, disparity patterns in AFR increased, except for education-related inequalities which varied based on various summary measures and contextual interpretation.

Similar with previous studies [35, 36], our study showed significant wealth-driven disparities from 2009 to 2016 disfavoring the poorest adolescents. While policymakers need to ensure equalizing opportunities for all, evidence suggests that economic growth benefits only a small advantaged elite, and that is factual of Timor-Leste [6, 37]. In this country, adolescent pregnancy is more likely to occur in vulnerable communities, mainly driven by poverty, sexual violence, lack of education and poor access to modern contraceptives [6, 9]. At the community level, adolescents in rich households are more likely to delay childbirth and achieve powerful levels of employment [38].

Our findings also suggest that there were wider education-related inequalities in AFR over the seven-year study period, although the pattern varied from decreasing (by complex measures) to constant (by simple measures). For instance, in 2016, AFR among non-educated adolescents was higher by 58.7 percentage points compared to adolescents who had secondary school and above level of education. The 2009 and 2016 AFR could be reduced by 33.8 and 25.8% respectively, if the country had avoided relative education related inequalities. Similarly, if the absolute education related disparities were avoided, it was possible to reduce the 2009 and 2016 AFR by 18.4 and 11.4 percentage points respectively.

Congruent with previous studies [39–41], we observed a lower risk of early childbearing with an increase in adolescent girls’ education. While the gaps in AFR between educated and uneducated narrowed over the seven-year study period, there is a need for much work to be done to prevent adolescent pregnancy and its adverse maternal and neonatal outcomes. Adolescents with lower levels of education are significantly less likely to be able to negotiate safe sex, and therefore higher educational attainment may increase their bargaining power, access to knowledge and resources, including decision-making autonomy [39]. A previous study in Norway shows that one additional year of schooling reduces the chances of adolescent pregnancy by eight percentage point [40]. Improvements in female education influences fertility because it provides an informed decision on the number of children a woman may have and the number of children she wants through uptake of availability of modern contraceptives and the knowledge on how to use them [41, 42].

Another trend in society is that many adolescent girls with unwanted pregnancies resort to clandestine abortion amid restrictive abortion law and, as a result, suffer serious complications and death [42, 43]. Despite this fatal possibilities, a large proportion of girls in these communities are sexually active, and many lack access to health services including contraception, safe abortion and skilled pregnancy care [42]. The situation is largely attributable to pervading socio-cultural beliefs and practices which stigmatize discussions on matters related to sex, sexuality, induced abortion and pregnancy in young people. The entrenched gender inequality and adverse social perceptions in predominantly rural communities that disadvantage young women as compared to boys have never been challenged as a social justice and equity issue that imperils the adolescent girls’ wellbeing [44].

Each year, there are an estimated 2.7 million un-intended pregnancies among adolescent women living in South Central and Southeast Asia [45, 46]. There were a total of 6.8 million unsafe abortions in South-Central Asia, 3.1 million in South-eastern Asia, and 830,000 in Western Asia in 2008 [46, 47], which accounts for 45.7% of all unsafe abortions among women aged 15–24 that occur yearly in the developing world [46, 48]. According to the World Health Organization, 12% of maternal deaths in the region are the result of unsafe abortion [46, 47]. In addition to social and mental health problem, unsafe abortion has serious complications for women, including the adolescents such as incomplete abortion, hemorrhage, infection, uterine perforation, damage to the genital tract and internal organs, which again make their life more complicated. There is also huge economical loss; The annual cost of treating major complications from unsafe abortion is estimated at USD 553 million [49].

Wide pro-urban disparities of AFR were identified with an increase in over time worsening of the gap between these two groups. From the 2016 survey, our study findings highlight that about 37.1 percentage points of AFR and this was higher among rural residents compared to their counter parts. In same survey year, The Ratio measure again tells us, AFR among rural adolescent were 2.8 times higher compared to urban adolescents. Similarly, the country could cut the 2016 AFR by 52.3% and 23.1 percentage points if the relative and absolute urban-rural disparities were avoided respectively.

This finding is consistent with previous studies that showed existence of urban-rural disparities in AFR [50–52]. The reason for lower AFR among urban adolescent girls could be related to accessibility and availability of contraceptives [52, 53], relatively low influence of socio-cultural factors, no geographic difficulty barriers and lack of barriers to contraceptive and other sexual and reproductive health services [54, 55]. Additionally, urban adolescents have no extreme shame or fear to use contraceptive related to privacy and permission issues [55]. Arguably, this could also be because adolescents from urban areas spend more years attaining higher educational level, thereby leading to a delay in getting married and having children compared with their counterparts from rural areas [56]. Also, young people living in urban areas have better access to the Internet, creating good opportunities to be exposed to sexual problems and to learn about risky sexual behavior [56, 57]. Evidence has shown that rural to urban migration has been major social phenomena in the country and this migration resulted in the 33% increase in the population of Dili, and in the 40% increase in the population of the country as a whole. Since migrants move in search of education and job opportunities in the urban areas, it is likely that these migrants would be those with lower fertility preference and consequently contribute to the overall lower AFR in urban areas. However, available literature suggest that rural to urban migration has been closely associated with kinship and broader affinal networks [58] and whether this rural to urban migration contributed to the lower AFR in urban areas of Timor-Leste needs more work.

Furthermore, these young people may use mass media as a primary source of information, particularly if it relates to issues of friendship, romantic relationships and sexuality [56, 57]. In Timor-Leste, AFR is generally higher in rural areas where early marriage is prevalent, especially among girls with less educational achievement and lower socio-economic status [6]. On this basis, given the role of socio-cultural standards in influencing sexual and health-seeking practices, there is the need to involve community memebers, especially gatekeepers such as parents and community leaders in the development and implementation of programmes aimed at dealing with the high rates of adolescent pregnancy in the country. Adolescent pregnancy can also be prevented by involving male or young male [59] as they can support by using male contraceptive [59, 60]. However, they may feel as this is their female partner sole responsibility [61, 62]. Most young men also have information deficit about contraception [62] even in developed nation like US [59]. Open sexual communication between men/young men and women can significantly reduce teenage pregnancy as it facilitate condom use [63, 64] or condom and other contraceptive use (dual method) [63]. Hence, policy makers and implementers such as educators and health care providers need to understand that it is highly helpful integrating male adolescents as one approach to prevent reduce teenage pregnancy [65].

Another interesting finding from this study was the significant AFR disparities across subnational regions that showed an upward pattern over 7 years. In the Oecussi region, for example, AFR was 60 percentage points higher than in Dili. The nation’s 2016 AFR would have reduced by 53.4% and 23.6 percentage points if the relative and absolute region-based disparities were avoided respectively. This is similar with previous study and could be related to magnitude of early marriage across the regions [12]. Early marriage disparities across regions could be a reflection of differences in the socio-cultural and economic situations of the regions, such as differences in religion, traditional norms and the lack of inter-regional uniformity in the implementation and enforcement of the marriage law, among other factors [12]. In Timor-Leste, a recent study found that gender norms and unequal power relations between girls and boys were key drivers of adolescent pregnancy [6, 9].

### Strengths and limitations

The study has several strengths. First, unless there are studies that were done but never published, to the best of our knowledge, our study is the first to examine inequalities in fertility rates among adolescents in Timor-Leste using two rounds of DHS data. Therefore, our findings can be useful in guiding both policy and future research on adolescent fertility rates in this country. Second, the adoption of several measures of inequality contributes to the quality of our results and the limitations of each measure are better complemented by the strengths of the others. Using both relative and absolute inequality measures in the same study has the potential to help investigate the magnitude and trend of inequality from various dimensions and perspectives. Thirdly, the study presented the inequality findings for each subgroup of the equity stratifers, and this can assist the government to identify where and how to focus their efforts towards realization of the equity-oriented sustainable development goal (SDG) in relation with adolescent health. Finally, the study used the high-quality data available through the WHO health equity monitor database which strengthens the quality of the conclusions drawn from the study. We are confident our findings reflect reliable evidence. Nonetheless, the study has some limitations. It focused on description of the nature of adolescent fertility rate inequality in light of the recommended dimensions of health inequality. Nevertheless, detailed identification of explanatory variables that underlie the detected disparity necessitates a decomposition technique. Future studies require to put on this statistical method to better recognize the contributions of each determinant factor on the observed AFR inequality using analytical approaches such as decomposition analysis. To end, DHS data are cross-sectional and so no causality can be inferred from the results. Also, the study used secondary data and so the authors had no influence over the selection and measurement of the variables. Finally, there is the likelihood of under-estimating AFR in the survey since some of the pregnancies that may have resulted in births would end up been terminated voluntarily by the adolescents.

### Policy and public health implications

Country-specific actions that can be done to reduce the socio-economic and geographical gaps in AFR may include evaluating inclusiveness or whether the national economic growth are benefiting those disadvantaged subgroups or not, rural-focus fertility control strategies such as awareness creation about fertility control and giving the service including outreach service in the place where the adolescents are available such as at rural school and market day need to be designed. Interventions such as schooling and asset-building for girls would be directed to very young adolescents in the crucial age range of 10 to 14 in order to counter pressures on girls to marry and bear children for social and economic security. It needs more attention and priorities for those younger adolescents as they are highly vulnerable and less mature in addition to them havingless coping capacities for discrimination related problems from their friends, families and communities at large [66].

## Conclusions

This study proves the continued inequality influencing adolescent fertility rate to the advantage of adolescent girls in better socioeconomic classes and who are located in urban settings, with rising up of disparity over the time period of the study. This study’s findings support available evidence suggesting that socioeconomic positions play key roles in disparities observed in AFR [11, 38, 41]. Policy makers need to stop worsening disparities by targeting subgroups which are highly disadvantaged. Without formulating a strategy that targets those subgroups (subpopulations at the lower end of the socioeconomic spectrum and those in rural areas), it is unlikely the country will avert inter-generational maternal and newborn morbidity and mortality. Potential psychological problems, chronic illness and deaths of both mothers and newborns could be stopped if more works are done on these underprivileged subpopulations. Lack of strategic planning hinders the achievement of SDGs in reducing maternal mortality among adolescents and neonatal deaths. To capacitate adolescents to decide about their sexual and reproductive health, socio-cultural problems like early marriage, stereotyping should be tackled since it leads them to be voiceless and do not exercise their right. To go further to sustainable development, it is highly valuable empowering girls and women economically and through education. Emphasis should be given to rural adolescents who have low contraceptive access either due to geographic or cost barrier.

## Data Availability

The datasets generated and/or analyzed during the current study are available in the WHO’s HEAT version 3.1 [https://www.who.int/gho/health_equity/assessment_toolkit/en/].

## Abbreviations

ACI: Absolute concentration index
AFR: Adolescent Fertility Rate
CI: Confidence Interval
D: Difference
DHS: Demographic and Health Survey
EA: Enumeration Area
HEAT: Health Equity Assessment Toolkit
IMR: Infant Mortality Rate
PAF: Population Attributable Fraction
PCA: Principal Component Analyses
PSUs: Primary Sampling Units
R: Ratio
SDGs: Sustainable Development Goals
WHO: World Health Organization
